# Nationwide data on home care and care home residence: presentation of the Swedish Social Service Register, its content and coverage

**DOI:** 10.1177/14034948211061016

**Published:** 2021-12-29

**Authors:** Anna C. Meyer, Glenn Sandström, Karin Modig

**Affiliations:** 1 Unit of Epidemiology, Institute of Environmental Medicine, Karolinska Institutet, Sweden; 2 Stockholm University Demography Unit (SUDA), Stockholm University, Sweden; 3 Department of Historical, Philosophical and Religious Studies, Umeå University, Sweden

**Keywords:** Health registers, register data, coverage, population, ageing, home care, elder care, Sweden, administrative registers

## Abstract

**Aims:**

All Swedish municipalities are legally obliged to provide publicly funded elder care to individuals in need. The Swedish Social Service Register collects data on such care. It is the only nationwide source of information on care home residency and use of home care but has rarely been used for research. This study aims to present the content and coverage of the Social Service Register and to provide guidance for researchers planning to use these data.

**Methods:**

For each month between 2013 and 2020, we examined which of Sweden’s 290 municipalities reported data to the Social Service Register. We calculated proportions of the population (restricted to ages 80–89 years to enable comparison) that were reported to the Social Service Register in each municipality and presented the types and amount of care recorded in the register.

**Results:**

The proportion of municipalities reporting to the Social Service Register increased from 82% to 98% during the study period but several municipalities reported fragmentarily and inconsistently, particularly during earlier years. Among municipalities reporting to the Social Service Register, 9% of the population aged 80–89 years resided in care homes and 19% received home care, but the registered amount and types of care varied substantially between municipalities and over time.

**Conclusions:**

**The Swedish Social Service Register provides valuable data for research on aging and elder care utilisation, but data should be selected and vetted carefully, especially for earlier years. The amount and types of care may not always be comparable between geographical regions and different time periods. In recent years, however, the coverage of the Social Service Register is good.**

## Introduction

Population-wide data on elder care are invaluable for research on aging and care use. Sweden, together with the other Nordic countries, has a long tradition of collecting nationwide data in administrative population registers. Through unique personal identity numbers assigned to each resident, these registers can be linked for research purposes. However, administrative registers are not created for research purposes and researchers should be aware of their content and potential pitfalls when using these data. The content and coverage of many such registers have therefore been described in previous studies [1–4]. The Register for Municipal Care of the Old and Functionally Impaired [Registret över insatser till äldre och personer med funktionsnedsättning enligt socialtjänstlag], in short the Social Service Register (SSR), contains information on older individuals’ care home residence as well as publicly funded home care. Held by the National Board of Health and Welfare, the SSR contains information about the amount and type of long-term care provided to older individuals on a monthly basis, and is the only nationwide register in Sweden recording care home residency. Despite its potential value, the SSR has only rarely been used for epidemiological, peer-reviewed research. In this study, we present the content, strengths and limitations of the SSR, and derive a number of recommendations for researchers aiming to use these data.

In Sweden, elder care is organised and financed by the municipalities; all Swedish municipalities are legally obliged to provide publicly funded elder care to older individuals in need according to the Social Service Act [Socialtjänstlag] [5]. These services can be provided by the municipalities themselves or contracted out by municipalities to private providers. All municipalities are also obliged to report provided care to the SSR. To allow older individuals to ‘age in place’, care is preferably provided in the form of home care services. These services include both personal care, such as help with showering or getting dressed, and practical help with household tasks, for instance cleaning or grocery shopping [5]. Only when a person’s needs can no longer be met in their own home are they transferred to care homes. Publicly funded care resources are allocated by the municipalities strictly based on needs assessments. Care is heavily subsidised and provided against small fees that are deemed universally affordable or even free for those with the lowest income. Although the uptake of privately paid elder care is increasing, publicly funded care in accordance with the Social Service Act still represents the vast majority of elder care in Sweden, and only a few older individuals purchase services on top of publicly financed care [6–8].

The SSR has colleced data on elder care since 2007, but data quality was limited in its earlier years which may have contributed to its sparse use in the scientific literature. The National Board of Health and Welfare has deemed the data quality sufficient to publish statistics based on the SSR for each year since 2007 (except for 2009), but previous research pointed out varying degrees of reporting to the register among Swedish municipalities [9]. Although reporting to the SSR is mandatory for all municipalities, its coverage and the amount of data reported appear to vary, which may have consequences for research. With this study, we aim to present the SSR, its content and coverage between 2013 and 2020 in order to inform researchers and facilitate its use for research on ageing, health and care use. We further present and compare the proportions of the older population reported to the SSR, the average amount and type of care registered, and the continuity of reporting in each of Sweden’s 290 municipalities to identify unreasonable reporting patterns that potentially indicate data limitations.

## Data and methods

### Structure of the SSR

Following a regulation of the National Board of Health and Welfare, all municipalities are obliged to report to the SSR on a monthly basis [5]. This includes all publicly funded care, regardless of whether home care or care homes are operated by the municipalities themselves or by private contractors. Moreover, the register neither records claims that have been submitted but not (yet) granted by the municipality nor granted claims that have not been utilised by the individual. The register may, however, contain claims that have only partially been utilised. For example, a person may not use the entire amount of home care they have been granted in each month. Data are entered electronically into a database by each municipality. The National Board of Health and Welfare collects and administers these data and hands the register out to researchers after ethical vetting. Basic quality control measures, for instance, the identification of missing or contradictory data, are in place at the National Board of Health and Welfare, and municipalities are contacted to complete or correct their data if necessary.

### Data sources and study population

The primary data source of this study is the SSR. We linked the register to a larger database, the Ageing and Health Cohort (AHC), which contains information about the total Swedish population to calculate the proportion of the total population aged 80–89 years that receives care according to the SSR in each municipality. Data were linked by Statistics Sweden through the personal identification number assigned to each resident of Sweden.

Although the SSR was already established in 2007, its structure changed substantially in 2012, rendering data recorded after 2012 not directly comparable to earlier data. In this work, we present the SSR from 2013 to December 2020, the last date for which data were available in the AHC. The SSR includes data on elder care and on long-term care for younger adults with physical, mental or intellectual impairment who receive assistance according to the Social Service Act. Here we focus on elder care and restrict our analyses to individuals over the age of 70 years.

### Analyses

A Swedish description of the variables included in the SSR together with guidelines for reporting has been published by the National Board of Health and Welfare [5]. We translated variable names and descriptions to English and present these together with their distributions in Table I.

**Table I.** Variables included in the SSR.

Variable name in the SSR	Definition	Distribution among records in the SSR^a^ *N*=19,984,030
Living arrangement *boform*	Permanently living in private residence, elder care home, or other living arrangement Private residence may in some cases include entirely paid-out-of-pocket service housing. Elder care home includes nursing homes and institutions that provide needs-tested and publicly funded elder care according to the Social Service Act. Other living arrangement includes those with no permanent residence, living in shelters, or other social housing arrangements	Private residence: 75.6% Elder care home: 24.2% Other living arrangement: 0.2% NA: 0.0%
Home care *htj*	Granted and executed home care claim in place on the last day of the month. May include individuals living in elder care homes	Yes: 69.5% No: 30.5%
Monthly home care hours *htjtim*	Number of granted home care hours per month. Up to 744 hours representing care around-the-clock	Available for 94.5% of records with home care
Short-term residence *korttid*	Temporary placement in medical or care facility for round-the-clock care (e.g. for temporary hospitalisation, rehabilitation, or relief of private caregivers) Duration of temporary placement recorded in variable *kortman*	Yes: 2.7% No: 96.6% NA: 0.6%
Daytime activities *dagv*	Social daytime activities for older individuals living in their own home that is publicly funded according to the Social Service Act (e.g. meetings to talk, play board games or go for a walk with others)	Yes: 3.1% No: 95.9% NA: 1.0%
Other support *abist*	Support within elder care not included elsewhere, e.g. providing necessary transportation	Yes: 0.8% No: 84.2% NA: 15.0%
Types of care included in home care claim		Distribution among records in the SSR^a^ with home care *N*=14,144,204
Service *htjserv*	Help with household chores (‘practical help’), e.g. cleaning, meal preparation, grocery shopping or running errands	Yes: 62.5% No: 31.9% NA: 5.6%
Personal care *htjpomv*	Support with all other tasks necessary to meet individuals’ social, physical and psychological needs, e.g. personal hygiene, getting dressed, eating or facilitating social contacts	Yes: 63.2% No: 31.1% NA: 5.7%
Social participation *htjled*	Support aimed at reducing social isolation and enabling participation in social activities outside the home	Yes: 7.4% No: 85.3% NA: 7.2%
Relief of private caregiver *htjavan*	Temporary provision of care to relieve private caregivers such as spouses	Yes: 1.2% No: 89.8% NA: 9.0%
Meal service *servmatd*	Indicates whether practical help recorded in variable *htjserv* includes the delivery of prepared meals (‘meals on wheels’)	Practical help provides only meal service: 2.3% Practical help provides meal and other services: 9.5% Practical help does not include meal service: 87.1% NA: 1.1%
Security alarm *pomvtrygg*	Indicating whether personal care recorded in variable *htjpomv* includes the installation of an alarm system to notify medical services in emergency situations	Personal care includes only alarm: 7.0% Personal care includes alarm and other care: 8.5% Personal care does not include alarm: 82.4% NA: 2.2%
Care provided based on separate claim (among individuals without home care or independently of the main home care claim)		Distribution among records in the SSR^a^ *N*=19,984,030
Meal service *matd*	Delivery of prepared meals (‘meals on wheels’)	Yes: 7.6% No: 80.9% NA: 11.5%
Security alarm *trygg*	Installation of alarm system to notify medical services in emergency situations	Yes: 46.2% No: 47.8% NA: 5.9%
Social participation *ledsag*	Support aimed at reducing social isolation and enabling participation in social activities outside the home	Yes: 3.0% No: 90.9% NA: 6.8%
Relief of private caregiver *avan*	Temporary provision of care and support to relieve private caregivers such as spouses	Yes: 1.2% No: 90.9% NA: 7.9%

Additional information collected in the SSR: personal number, municipality, month and year, specific support for individuals with mental disabilities in their own home (applicable to 0.4% of records).

NA: not available (missing); SSR: Social Service Register.

a All monthly records in the SSR during January 2013 to December 2020 among individuals aged 70 years and older in the Ageing and Health Cohort. As data are available on a monthly basis, one record in the SSR pertains to one person during one month. Individuals receiving care for longer time periods therefore appear as several records in the SSR.

We examined several indicators related to the coverage of the SSR. First, we examined for each month separately whether municipalities reported to the SSR. Next, we calculated the proportion of the older population residing in each municipality that were reported to live in care homes or receive home care, respectively. To account for regional differences in age distributions, we restricted these analyses to the age group 80–89 years, which includes the majority of care recipients. Although there are likely to be differences between municipalities, the proportions of care recipients in this age group should be reasonably similar in all municipalities and relatively stable over time. Very large deviations from the average and sudden fluctuations could potentially indicate data problems that should be acknowledged.

Care home residence was identified through the variable *boform* and receiving home care was defined as care registered in the main home care variable *htj* (see Table I), regardless of which types of home care were registered in other variables in the SSR. The proportion residing in care homes was calculated as the number of individuals living in an elder care home (‘*särskild boende*’) according to the SSR at the end of each month divided by the municipalities’ population in the same age group during that month. Short-term stays in medical or rehabilitation facilities were not considered care homes. The proportion of individuals with home care was calculated as the number of individuals receiving home care divided by the population of the municipality living in their own home (i.e. not in care homes). Our calculations are based on the assumption that individuals receive care within the municipality they reside in. As elder care constitutes a financial burden to municipalities, this assumption is likely to be violated only in exceptional cases.

Moreover, among home care recipients, we calculated the mean number of granted home care hours for each municipality and month (variable *htjtim*). To examine the impact of outliers, we further present mean hours excluding municipalities that reported unreasonably high (mean above 200 hours) or low (mean below 5 hours) values (13.8%). In a final step, we additionally excluded small municipalities which reported less than 100 home care recipients at least once during the study period (56.4%).

Finally, we examined the types of home care reported to the SSR. We calculated the proportion of home care recipients who received the two main types of home care; that is, practical help and personal care (variabes *htjserv* and *htjpomv*). As in small samples, proportions are more likely to be influenced by chance, we excluded months in which municipalities reported less than 100 individuals in the age group 80–89 years to the SSR (28.6%). Next, we present the proportion of home care recipients who receive the remaining home care types; that is, social participation, relief of kin caregivers, meal service and security alarm. Some individuals may receive these services; for instance, the installation of a security alarm, without being granted any formal home care. Each of these types of care are, therefore, recorded in two different variables reflecting (a) care given as part of an individuals’ granted home care claim (variables *htjled, htjavan, servmatd, pomvtrygg*) and (b) care given independent of any home care claim (variables *matd, trygg, ledsag, avan)*.

### Continuity of reporting

Sudden and considerable changes in the number of care recipients within a municipality may indicate unreliable data or changes in reporting procedures. Therefore, we calculated monthly changes in the number of care recipients between two successive months excluding months in which municipalities did not report to the SSR. An arbitrary cut-off value of 30% was chosen to define large variations between two successive months.

### Ethical approval

This study was approved by the regional ethics committee in Stockholm (permit numbers Dnr 2011/136-31/5 and Dnr 2017/2518-32). The board waived the need for patient consent.

## Results

### Variables in the SSR

Table I provides an overview of variables included in the SSR. The register records whether a person receives any home care, the number of granted hours per month, and the types of care they receive. The two primary types of home care are practical help with household chores and personal care; for instance, support with showering or getting dressed. In addition, the SSR distinguishes between four further types of care; namely, support to enable social participation, relief of private caregivers, meal service and security alarm (see Table I for descriptions). Each beneficiary can receive home care for one or several of these purposes.

Support to enable social participation, relief of private caregivers, meal service and security alarm may also be provided independently of receiving home care; that is, among individuals who do not receive home care or according to separate claims. This implies that a person could have a valid home care claim that does not include meal service and an additional claim that does include meal service. As a result, researchers studying, for example, meal services should take into account two variables, namely *servmatd* (meal service as part of home care) and *matd* (meal service independent of home care). In addition to granted home care and residence in care homes, the SSR contains information on temporary placements in medical and care facilities, participation in daytime social activities, or other support funded by the municipalities in accordance with the Social Service Act.

### Missing data

The amount of missing information varies between variables included in the SSR (Table I). Data on living arrangements and whether or not a person receives home care is available for virtually all records in the SSR. The number of home care hours is available for 95% of home care recipients and the availability of information on home care types ranges between 91% for the relief of private caregivers and 99% for meal service.

### Coverage and proportion of the population reported to the SSR

**Figure 1:**
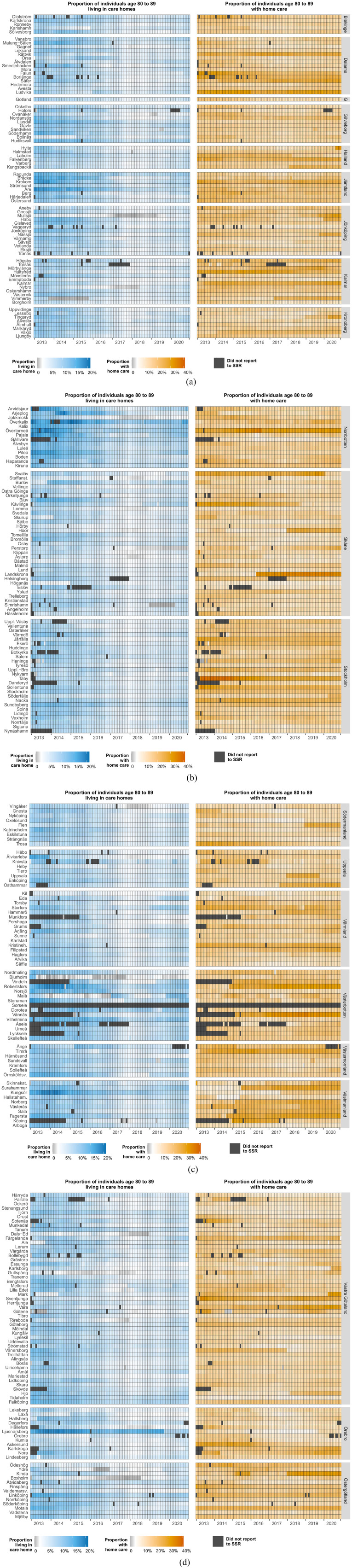
Reporting to the SSR and the proportion of 80–89-year-old residents receiving home care and living in care homes by municipality during 2013 to 2020. Proportion receiving home care restricted to individuals living in their own home. SSR: Social Service Register.

Despite legal obligations, not all municipalities reported consistently to the SSR. The proportion of municipalities reporting at least one person to the SSR increased over time from 84.5% in January 2013 to 99.7% throughout 2019, and subsequently decreased to 98.3% in December 2020 (Supplemental Figure 1). Reporting for each of Sweden’s 290 municipalities is shown in Figure 1 with dark grey-shaded areas representing months in which no data were reported to the National Board of Health and Welfare. In total, 163 municipalities (56.2%) reported to the SSR consistently, and one municipality did not report to the SSR in any month. Spells of non-reporting were often short and more likely to occur at the beginning of the study period. Disregarding short spells of non-reporting of 1–2 months and the time period before January 2014, 269 municipalities (92.8%) reported consistently to the SSR.

### Proportions receiving home care and living in care homes

In municipalities reporting to the SSR, on average 4.1% of individuals over the age of 70 years lived in elder care homes and 9.1% received home care while living in their own home. The mean age of individuals reported to the SSR was 84 years. Among individuals aged 80–89 years, 6.9% lived in elder care homes and 17.0% received home care.

Figure 1 shows the proportions of 80–89-year-old individuals living in care homes and receiving home care by municipality and month, indicating considerable regional and temporal variation. Excluding municipalities and months which reported fewer than 100 individuals to the SSR, the proportion of individuals aged 80–90 years in care homes ranged from 3.0% (5th percentile) to 12.3% (95th percentile) and the proportion of individuals receiving home care from 10.2% (5th percentile) to 27.1% (95th percentile). A spreadsheet containing these proportions for each municipality and month is available in the supplemental materials (Supplementary File 1).

### Amount and type of home care

**Figure 2.**
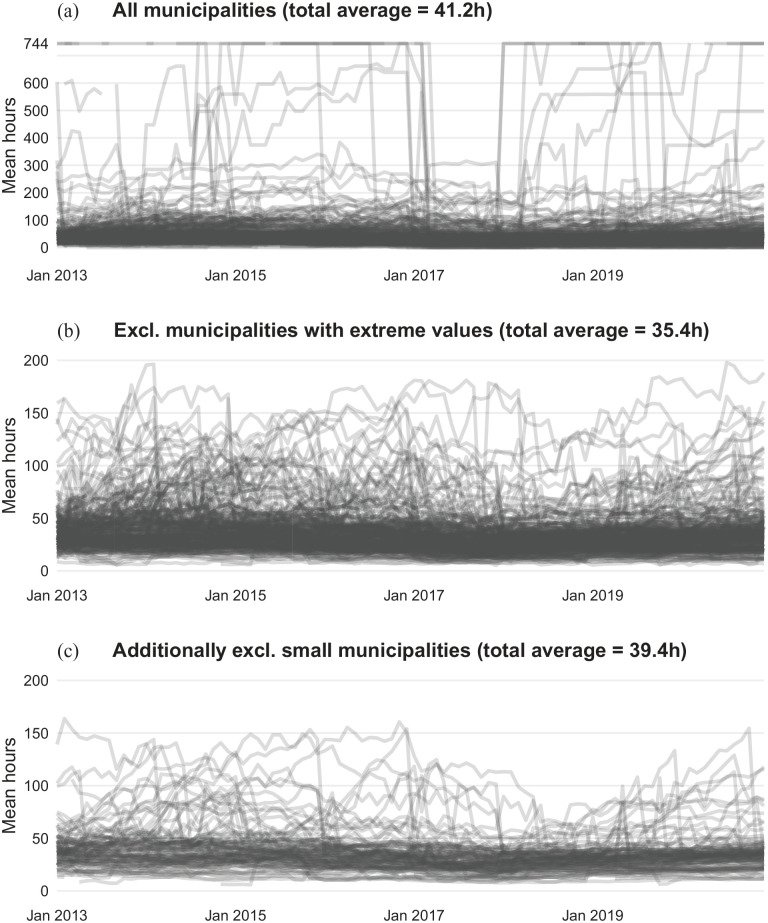
Mean number of granted home care hours by municipality among individuals aged 80–89 years, January 2013 to December 2020; 744 hours indicate around-the-clock home care. (a) All 290 municipalities in Sweden. (b) All municipalities excluding those that reported mean numbers below 5 or above 200 hours for at least one month (*N*=237). (c) Additionally excluding municipalities with less than 100 home care recipients in at least one month (*N*=144). List of municipalities included in (b) and (c) provided in Supplemental File 1. Municipalities with missing data in some months are shown with gaps. SSR: Social Service Register.

On average, home care recipients were granted 41.2 hours of home care per month; that is, approximately 10 hours per week, but the average number of granted home care hours varied across municipalities and over time (Figure 2). Most municipalities reported on average between 20 and 50 home care hours per recipient, while some reported strikingly high numbers, sometimes exceeding several hundred hours per person. Even when excluding smaller municipalities and those that reported very large or small numbers, there were considerable differences in the average number of granted home care hours between municipalities (Figure 2c).

**Figure 3.**
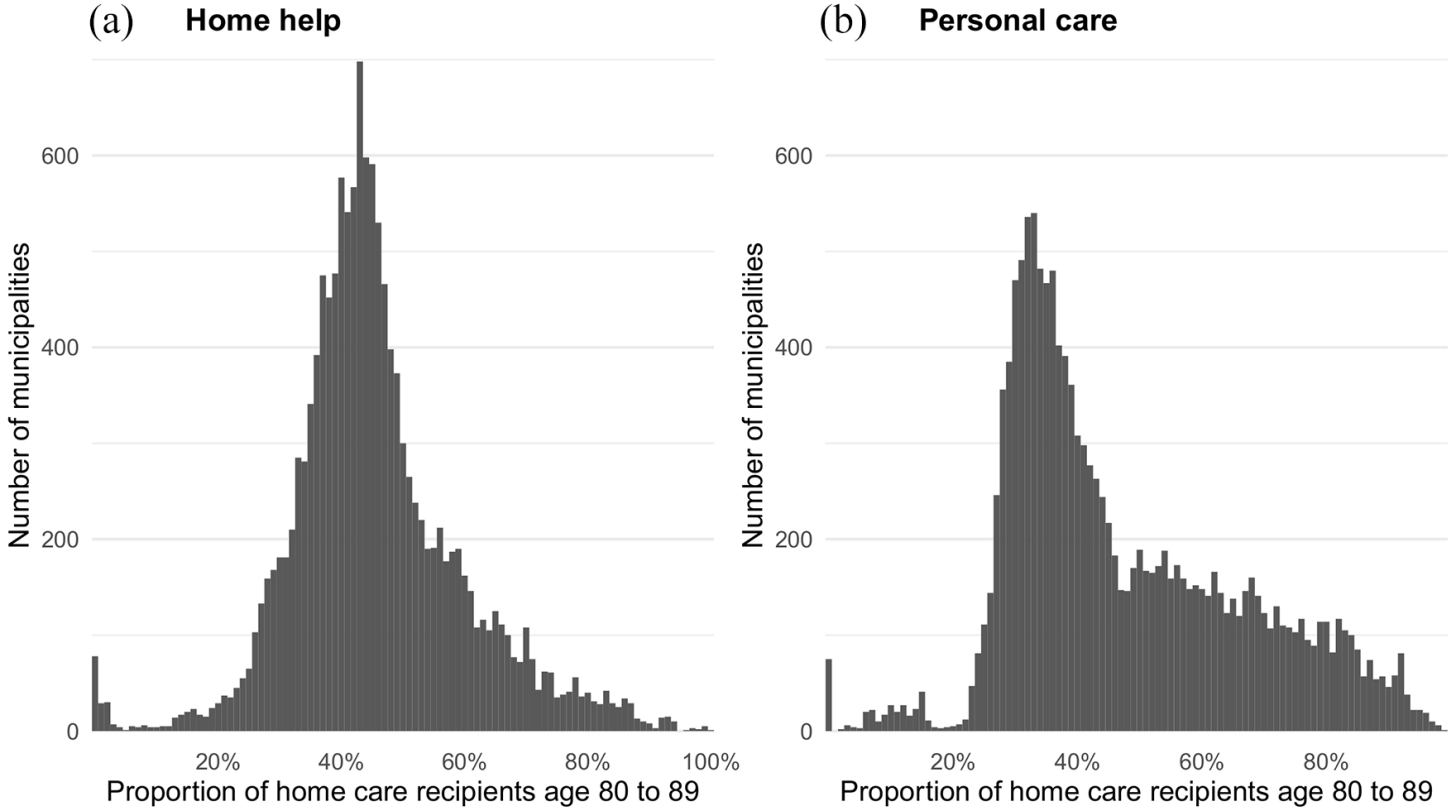
Proportion of 80–89-year-old individuals in the SSR receiving (a) practical help or (b) personal care. Histograms include monthly observations (*n*=19,872) from 207 municipalities. List of included municipalities provided in Supplemental File 1. Months during which municipalities reported did not report to the SSR are not shown. SSR: Social Service Register.

The proportions of home care recipients receiving the two main types of home care differed between municipalities (Figure 3). On average, municipalities reported practical help and personal care for approximately 40% of home care recipients, but proportions varied substantially. Proportions between 30% and 70% were common, with several municipalities reporting even lower or higher proportions (Figure 3). There were no apparent differences between time periods, indicating increasing or declining proportions of individuals receiving practical help or personal care (data available in Supplemental File 1).

**Figure 4.**
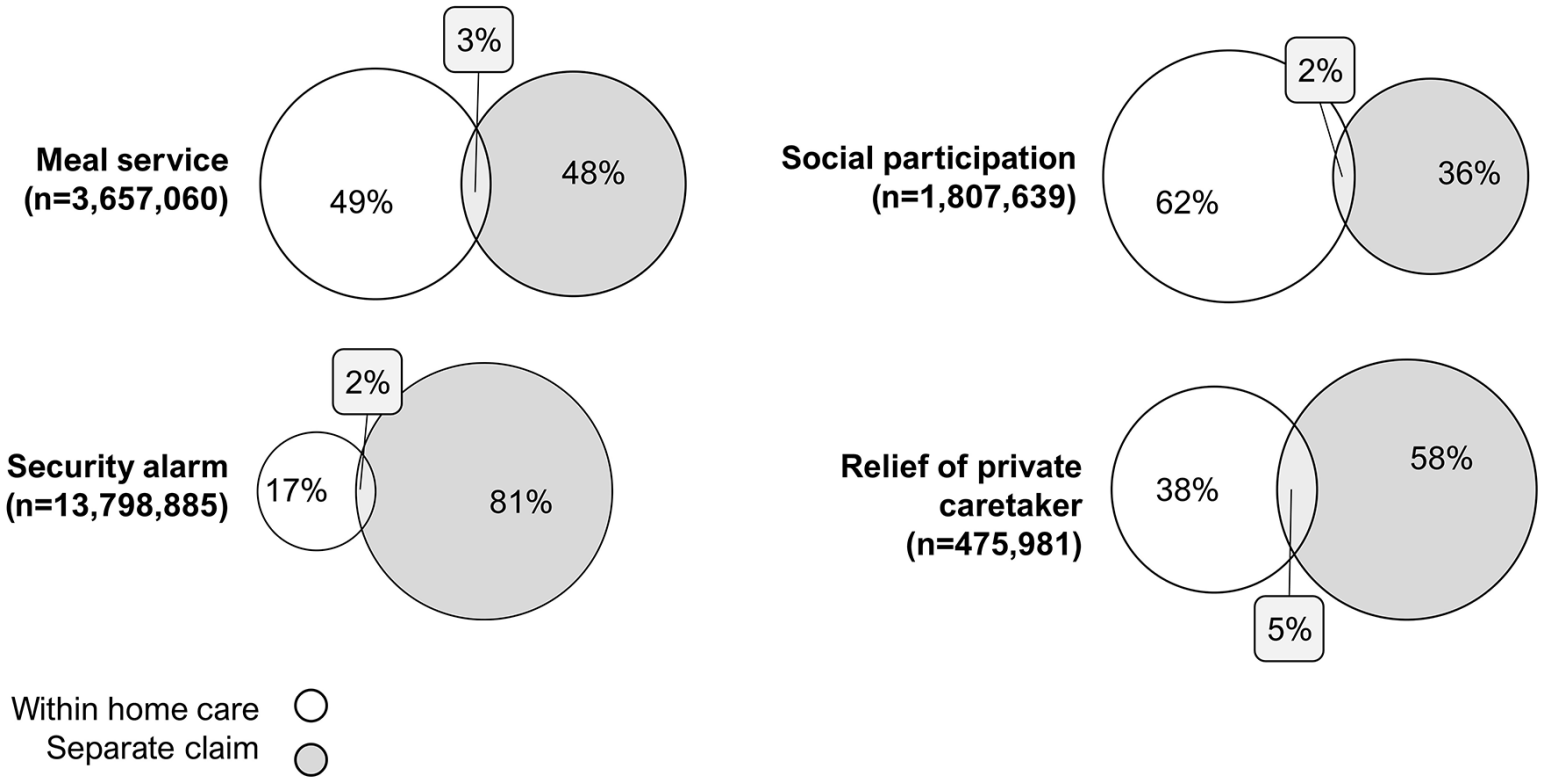
Proportion and overlap of records in the SSR receiving the same type of care as part of home care claim and according to a separate claim. Calculated for all monthly records in the SSR during January 2013 to December 2020 among individuals aged 70 years and older in the Ageing and Health Cohort. SSR: Social Service Register.

Figure 4 shows the number of records in the SSR receiving meal service, security alarm, support to enable social participation and relief of private caregivers, as well as the proportions receiving these types of care as part of a home care claim and according to separate claims. Security alarm is the most common of these care types and is often provided according to separate claims. Between 2% and 5% of records indicate that types of care were given both within and in addition to a person’s home care claim.

### Continuity of reporting

Monthly changes in the number of individuals reported to the SSR between two successive months are available in Supplemental File 1. We observed at least one change exceeding 30% among 43.8% of municipalities.

## Discussion

The Swedish SSR provides valuable data for research on care use among the old, but researchers should be aware of its structure and potential limitations; for example, regarding its coverage during earlier years, before starting to use the data for research purposes. Our analyses indicate that several Swedish municipalities report fragmentarily and inconsistently to the register, and data should therefore be selected and vetted carefully. However, our data also show that the coverage of the register across municipalities improved substantially over time and is overall good for the most recent years.

Data in the SSR suggest that the shares of older individuals who receive home care or who live in elder care homes differ considerably between Sweden’s 290 municipalities, as do the average number of home care hours per person and the types of home care provided. It cannot be determined in this study to what extent these observed differences reflect differences in care needs between municipalities, disparities in access to home care and care homes, or differences in reporting to the SSR. Even if the Social Services Act regulates that all municipalities must provide care to older adults in need, Swedish municipalities have a high level of autonomy in how the care is organised, have different economic resources, and varying degrees to which they prioritise geriatric care compared to other social services. One might argue, however, that the magnitude of variation between municipalities and – in some municipalities – over short periods of time indicates differences in recording or reporting practice rather than actual differences in provided care. It is possible that the definitions of variables reported to the SSR are unclear to some care providers or that the resources to ensure a thorough and consistent reporting are lacking. As a result, the comparability of data between geographical regions and time periods may be limited. Further studies are needed to investigate to what extent data on care reported to the SSR reflects individual health and care needs. The specificity of measuring care needs through the SSR is likely to be higher than the sensitivity, meaning that home care and care home residency can be used as a proxy for poor health among individuals receiving such care, whereas the absence of care can not necessarily be seen as a proxy for being healthy. However, although a person’s health and care dependency are probably strongly correlated, they may not always correspond. In some cases, individuals may be care dependent but can still be considered healthy. While our study examined the consistency of reporting to the SSR among the Swedish municipalities, further research is also needed to examine the consistency of SSR data at the individual level.

Despite this, data in the SSR may be useful for many research questions, particularly when excluding some municipalities with limited reporting and when focussing on recent time periods during which reporting became more consistent. It is the most comprehensive source of information regarding older adults’ living arrangements for the total population in Sweden. The increasing coverage of the SSR indicates improving data quality over time and, oftentimes, gaps in reporting are limited to one or 2 months. Imputation using data from successive months may be a strategy to eliminate such shorter gaps. It should be noted that the older population in some municipalities, particularly in the counties Västerbotten and Norrbotten, is small which may increase variation due to chance. However, even in the smallest municipality, there were at least 179 individuals aged 80–89 years in each month. In addition, part of the regional differences presented in this study probably reflect disparities in health and care needs in the population rather than inconsistent reporting to the SSR. Previous research indeed supports the fact that regional disparities in home care use between Swedish municipalities are largely explained by actual differences in care needs in the population [10]. Therefore, some differences between municipalities are to be expected and may not be attributable to under or over-reporting.

The SSR contains data on publicly funded, needs-tested elder care in Sweden, which can be operated by the municipalities themselves or contracted out to private providers paid by the municipalities. It is known that the proportion of private elder care providers is increasing and varies between Swedish municipalities [8, 11] but it remains unknown whether and how this may affect data quality in the SSR. Care that is paid entirely out of pocket is not registered in the SSR. Although older individuals have financial incentives to utilise municipal care, it is possible that some opt for privately paid services, especially in bigger cities where there is a market for such services. However, the majority of people who need help with personal care probably receive social services provided by the Swedish universal system, which can be identified in the SSR. Researchers should also keep in mind that informal support provided by close kin, most often spouses or children, is increasing [7, 12].

Home care is currently the backbone of Swedish elder care. Its utilisation is determined by older adults’ health and care needs but may also have significant effects on older individuals’ health and wellbeing. It is therefore important to make use of the data in the SSR and continue to conduct research exploring these data. Furthermore, the National Board of Health and Welfare reported that the coverage of many quality registers of health and care in old age depends on older individuals’ living situations; that is, differs between community-dwelling individuals and those living in care homes [13]. This emphasises the importance of reliable data on older adults’ living situations. In this study, we presented and investigated data in the SSR and developed several suggestions for researchers using these data. In summary, researchers using data from the SSR may consider disregarding the earlier time periods, particularly before 2014. Imputing data for months during which municipalities did not report to the SSR may increase the continuity of data. Nevertheless, even if data were consistently reported, municipalities included in a study should be selected carefully as data on the amount and types of care may not always be comparable. And finally, data from two different variables should be used to examine the prevalence of some types of care; for instance, security alarm. These suggestions may not be applicable to all research questions, and the actual strategy for handling data from the SSR depends strongly on specific research aims and methods. Considering the continuing population aging, the changing structure of Swedish elder care, and the invaluable information recorded in this nationwide register, we suggest the National Board of Health and Welfare should prioritise improving and validating data quality in the SSR.

## Supplemental Material

sj-xlsx-1-sjp-10.1177_14034948211061016 – Supplemental material for Nationwide data on home care and care home residence: presentation of the Swedish Social Service Register, its content and coverage Click here for additional data file. Supplemental material, sj-xlsx-1-sjp-10.1177_14034948211061016 for Nationwide data on home care and care home residence: presentation of the Swedish Social Service Register, its content and coverage by Anna C. Meyer, Glenn Sandström and Karin Modig in Scandinavian Journal of Public Health
